# Persistence of Infectious Human Norovirus in Estuarine Water

**DOI:** 10.1007/s12560-023-09577-w

**Published:** 2024-01-02

**Authors:** Daniel Rexin, Andri T. Rachmadi, Joanne Hewitt

**Affiliations:** Institute of Environmental Science and Research Ltd. (ESR), Porirua, 5240 New Zealand

**Keywords:** Norovirus persistence, Human intestinal enteroids, Shellfish, Estuarine, Seawater, Water quality

## Abstract

Norovirus is the predominant cause of viral acute gastroenteritis globally. While person-to-person is the most reported transmission route, norovirus is also associated with waterborne and foodborne illness, including from the consumption of contaminated bivalve molluscan shellfish. The main cause of shellfish contamination is via the bioaccumulation of norovirus from growing waters impacted by human wastewater. However, data on the persistence of infectious norovirus in the environment are limited due to a lack of a human norovirus culture method in the past. In this study, we applied the recently established method of norovirus replication in human intestinal enteroids to determine the persistence of norovirus in artificial estuarine water at 25 ppt for up to 21 days at 4 °C and 16 °C in the dark. Infectious norovirus was detected for up to 21 days. The relative infectivity declined from 100 to 3% at day 21, with decay rate constants of 0.07 day^−1^ at 4 °C and 0.17 day^−1^ at 16 °C. There was no decrease in norovirus titres as measured by reverse transcription-droplet digital PCR (RT-ddPCR), confirming the lack of the relationship between norovirus infectivity and direct detection by PCR. The results confirm that norovirus can remain infectious for at least 3 weeks in an estuarine water environment, presenting associated health risks.

## Introduction

Norovirus is the predominant cause of viral acute gastroenteritis globally, causing a total cost of $ 4.2 billion to society per year (Ahmed et al., 2014). While person to person is the most reported transmission route, norovirus is also associated with waterborne and foodborne illness including via the consumption of contaminated bivalve molluscan shellfish, such as oysters and mussels (de Graaf et al., 2016; Mathijs et al., 2012). Norovirus presents a significant health risk to shellfish consumers and an economic risk for the shellfish producing industry.

The main cause of shellfish contamination is via the bioaccumulation of norovirus from growing waters impacted with untreated or inadequately treated wastewater, or from other sources such as septic tank run-off or boat discharges. To assess the risk of contamination, understanding the persistence of infectious viruses in the environment is an important factor (Campos et al., 2014). While other studies have demonstrated that enteric viruses such as enteroviruses can remain infectious for weeks at temperatures of 15 °C or lower, data on norovirus in the environment are limited (Bae & Schwab, 2008; Knight et al., 2013). The lack of a reliable norovirus culture method, up until recently, has meant that persistence data have relied mainly on volunteer and epidemiological studies, use of surrogate viruses and/or direct PCR assays. A volunteer study showed that norovirus remained infectious for at least 61 days in a spiked groundwater at room temperature in the dark (using an original dose of ~ 6.5 × 10^8^ genomic equivalent copies) but was limited due to the cohort size (Seitz et al., 2011). Surrogate viruses such as murine norovirus, feline calicivirus and Tulane virus exhibit different sensitivities to inactivation, disinfection and/or anti-viral agents (Augsburger et al., 2021; Knight et al., 2016). Data from only molecular testing methods, including those utilising an intercalating dye such as propidium monoazide may not accurately inform on infectivity, as results depend largely on the mode of inactivation (Leifels et al., 2020). A meta-analysis study revealed that following physical and disinfectant treatments, the reduction in human norovirus measured by molecular methods was less compared to the reduction of viral surrogates (Knight et al., 2016).

In 2016, replication of human norovirus using human intestinal enteroids (HIE) was first reported (Ettayebi et al., 2016). While a significant breakthrough, the main limitations, besides the cost and being labour intensive, remain the high variability of viral replication and uncertain relationship between the norovirus titre in the inoculum of the HIE culture and subsequential level of replication, and low method sensitivity (Estes et al., 2019). A subsequently published study describing norovirus replication in a salivary gland cell line also reported a similar low sensitivity (Ghosh et al., 2022). Despite these limitations, a few studies using HIE for norovirus persistence in waters have been reported (Desdouits et al., 2022; Kennedy et al., 2023; Shaffer et al., 2022). The goal of our study was to further expand the knowledge on norovirus persistence in estuarine water with an improved use of HIE culture for quantitative assessment of infectivity.

**Fig. 1:**
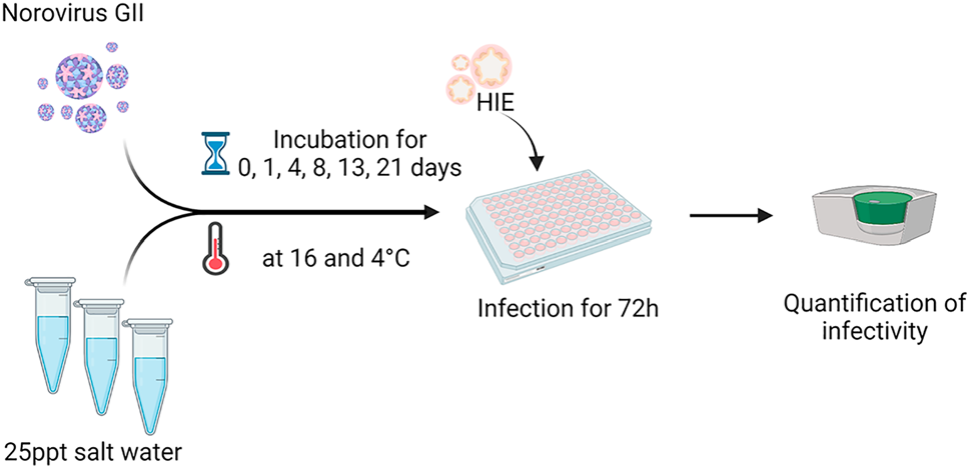
Depiction of experimental workflow. Created with BioRender.com

The aim of this study was to improve on the understanding of risks associated with human norovirus contamination in shellfish, and to specifically address the lack of data on the persistence of infectious norovirus in the water environment, specifically in estuarine water where oysters are cultivated. For this study, HIE cell culture was used to evaluate norovirus infectivity for up to 21 days in artificial estuarine water at 4 °C and 16 °C in the dark. The 16 °C corresponds to the average daily growing water temperature from Autumn (Fall) to Spring in temperate regions, with 4 °C used as a temperature control. To represent brackish water as in estuarine water, artificial water with a salinity of 25 ppt was selected (Whitfield & Elliott, 2011). To markedly improve sensitivity of the cell culture assay, the initial norovirus inoculum was concentrated to a very high viral titre and multiple technical replicates applied for each biological replicate (Fig. 1).

## Materials and Methods

### Norovirus

Faecal specimens submitted to the Institute of Environmental Science and Research for norovirus genotyping were used for this study (HDEC ethical approval number 18/NTA/193). To prepare a norovirus suspension, norovirus GII.4[P16] positive samples were pooled and resuspended in phosphate-buffered saline (PBS), pH 7.2 (BR0014G, Thermo Fisher Scientific) to give a 10% w/v suspension (400 mL total volume). The suspension was filtered through a 0.22 µm membrane (566–0020, Thermo Fisher) and concentrated to 8 mL by the addition of polyethylene glycol (PEG) 6000 (81260, Sigma–Aldrich) to give a final PEG concentration of 10%. This was followed by gentle mixing at 4 °C for 2 h, centrifugation at 10,000×*g* for 30 min, removal of the supernatant and final elution of the pellet in PBS. The suspension was aliquoted and stored at -80 °C until use. The concentration of norovirus in the resuspended concentrate was quantified using reverse transcription-droplet digital PCR (RT-ddPCR) following RNA extraction, as described below.

### Artificial Estuarine Water

To simulate estuarine water for increased reproducibility and to overcome the issues associated with site, intertidal and seasonal variability, for this study, artificial estuarine water rather than a filtered environmental water was used. Seawater salt (35001400, Instant Ocean Sea Salt) was resuspended with sterile reverse osmosis water to give a salinity of 25 ppt to represent a polyhaline salt concentration found in estuarine water (pH 7.4, not adjusted) (Whitfield & Elliott, 2011). Aliquots were stored at 4 °C until use.

### Experimental Setup

Aliquots of the water samples were inoculated with 10% v/v of norovirus suspension (total volume 1.2 mL) to a final concentration of ~ 5 × 10^9^ genome copies (GC) per mL and incubated statically at 4 °C and 16 °C in the dark for 0, 1, 4, 8, 13 and 21 days. Three sample replicates per time point were used. Following the incubation period, PEG precipitation was used to further concentrate norovirus to a final volume of 100 µL (i.e. tenfold volume concentration) as described above. Samples were then analysed using the HIE cell culture infectivity assay (Ettayebi et al., 2016). Viral RNA was also extracted from the concentrated water samples and analysed by RT-ddPCR to determine GC/mL at each time point. To avoid freeze-thawing samples for each time point, sample inoculation was planned so that all analysis (cell culture and direct RNA extraction) for all six timepoints was done on the same day.

### HIE Cell Culture and Infection

The HIE cell line was provided by Mary Estes (Baylor College, US) and cultures maintained as previously described (Ettayebi et al., 2016). Briefly, undifferentiated enteroids were propagated in Matrigel (CLS356231, Corning) and IntestiCult™ Intestinal Organoid Culture Media (INTp; 06010, Stemcell Technologies Ltd). For the infection, cells were seeded as monolayers and differentiated in 1:1 mix of Complete Media without Growth Factors (CMGF^−^) and component A of INTp media (INTd) for 5 days as described by Ettayebi et al. (2016). Cells were washed with CMGF^−^ media before infected with 5 µL of the concentrated sample diluted in 95 µL INTd media. Each sample was inoculated into ten wells of a 96-well plate (infection replicates) for the infection assay and incubated for 60 min at 37 °C. The cells were washed three times with CMGF^−^ media, followed finally by the addition of 100 µL INTd media. A baseline sample was stored immediately at − 80 °C and ten infection replicates per water sample were incubated at 37 °C in 5% CO_2_ for 72 h. Samples infected without inoculum and with 5 µL concentrated stool filtrate were included as negative and positive controls, respectively, and added per assay plate. Following HIE cell culture, samples were frozen and thawed three times to disrupt cells and centrifuged at 1000×*g* for 1 min.

### RNA Extraction

For each sample, viral RNA was extracted from a 50 µL aliquot of cell culture using the Magnetic Beads Virus DNA/RNA Extraction Kit (MV480, Geneaid Biotech Ltd, Taiwan) following the manufacturer’s instructions, and eluted to 30 µL. For direct quantification of norovirus (i.e. no HIE culture), viral RNA was extracted from 50 µL samples following PEG concentration. RNA was stored at − 80 °C until ddRT-PCR. Norovirus positive samples and negative (RNase/DNase free water) samples were included as extraction controls.

### Norovirus Quantification Using ddRT-PCR

Norovirus GII RNA was detected and quantified using One-Step RT-ddPCR Advanced Kit for Probes (1864022, BioRad Laboratories, Hercules, CA, USA), using primers and probes as previously described (Loisy et al., 2005). Each reaction consisted of 5 µL RT Supermix, 2 µL Reverse Transcriptase, 1 µL 300 mM DTT, 900 nM each primer (QNIF2: ATGTTCAGRTGGATGAGRTTCTCWGA, COG2R: TCGACGCCATCTTCATTCACA, BioSearch Technologies, CA, USA), 250 nM QNIFs probe (AGCACGTGGGAGGGCGATCG-FAM, BioSearch Technologies), 6.9 µL RNase/DNase free water and 2 µL extracted RNA. Droplets were generated from 20 µL of the reaction mix. This was followed by RT-PCR consisting of 50 °C for 60 min, 95 °C for 10 min, repeated cycling (45 × times) of 95 °C for 30 s and 55 °C for 60 s, 98 °C for 10 min, and final hold of 4 °C for 30 min using a Bio-Rad C1000 Touch thermal cycler (BioRad Laboratories, Hercules, CA, USA). Following the cycling, the droplets were read using the QX200 Droplet Generator (BioRad Laboratories) and data analysed with QX Manager software (version 1.2-STD, BioRad Laboratories). The norovirus copy number per RT-ddPCR reaction was converted to GC per mL sample. No template controls (2 µL RNase/DNase free water) were included on each assay plate. Samples were considered negative if there were less than three positive droplets. Viral RNA and controls were tested at least in duplicate.

### Data Analysis

Following HIE cell culture, norovirus replication was categorised into a binary data format, with samples considered infectious if the norovirus titre increased during the 72 h incubation time compared to the baseline sample at 1 h post infection (> × 1 of the baseline sample). The relative infectivity (%) was expressed as the proportion of infectious samples of the ten repeated infections. Data distribution was tested using the fitdistrplus package and regression models were approximated in R (Delignette-Muller & Dutang, 2015; R Core Team, 2018). The exponential model represented the best fit (data not shown), which was subsequentially fitted to each data set to infer an infectivity decay rate constant using following equation:

$${I}_{t}={100}^{\left(-\mathrm{K }\times \mathrm{ t}\right)}$$

where relative infectivity at time t is I_t_, the decay rate constant is *k*, and time is *t* in days.

Two-way ANOVA was used to assess significance with a *P* value of < 0.05 considered statistically significant. Data visualisation and statistical analysis was performed using Prism (GraphPad, Version 9.3.1).

## Results

### Direct Detection by ddPCR

**Fig. 2:**
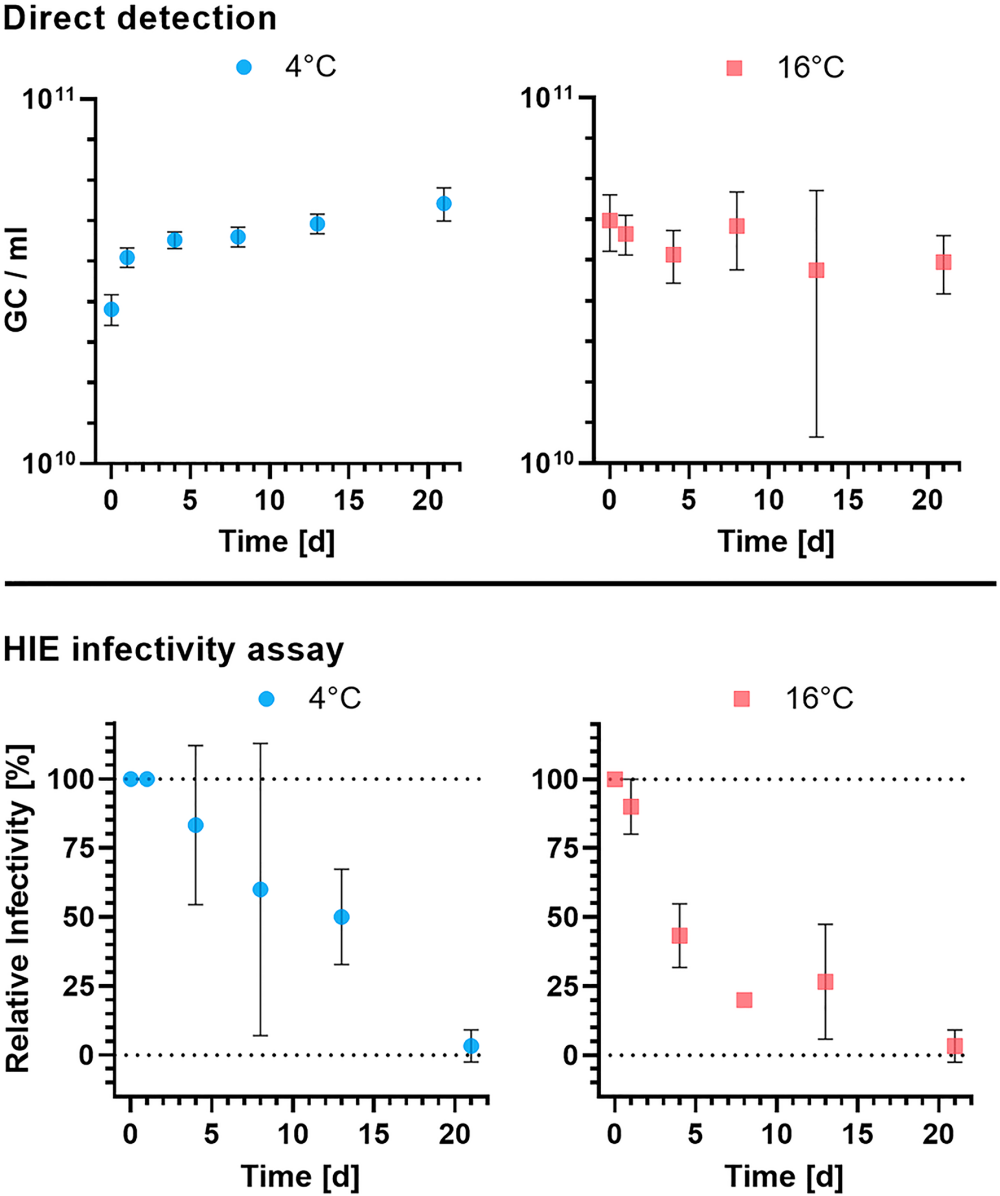
Decay of norovirus in artificial estuarine water at 4 °C (blue) and 16 °C (red) with three replicates per timepoint (days, d) and temperature. Top: Direct detection of Norovirus RNA by RT-ddPCR. Water samples were tested following PEG concentration. Mean concentration shown in GC / ml. Error bars indicate standard deviation. Bottom: Norovirus infectivity tested by HIE culture. Samples were tested for infectivity by HIE culture. Data points indicate mean of relative infectivity (fold-increase >  × 1) and error bars show the standard deviation (Color figure online)

The direct quantification of norovirus RNA by RT-ddPCR showed no significant difference of norovirus levels in samples over time (ANOVA, *P* value = 0.158, *α* = 0.05) and temperature (ANOVA, *P* value = 0.221, *α* = 0.05) during the duration of the experiment (Fig. 2).

### Infectious Norovirus Quantification by HIE Cell Culture

Infectious norovirus was detected throughout the length of the experiment, up to and including day 21 (Fig. 2). The relative infectivity declined from 100% at the start to 3% at day 21. When applying an exponential regression to the data, infectivity decay rate constants of 0.07 day^−1^ (95% confidence interval (CI): 0.04–0.10 day^−1^) for 4 °C and 0.17 day^−1^ (95% CI: 0.12–0.22 day^−1^) for 16 °C were determined. An extra sum-of-squares F-test resulted in a significant difference between the decay rates (*P* value = 0.0061, *α* = 0.05).

## Discussion

In our study, we applied HIE cell culture to address knowledge gaps on the persistence of norovirus infectivity in estuarine water. Our results demonstrate that infectious norovirus persisted for at least 21 days in artificial estuarine water at 25 ppt under laboratory conditions at 4 °C and 16 °C. While the levels of infectious virus were at the lowest detectable level at day 21, the limited sensitivity of HIE assay of ~ 1000 genome equivalents per infection (well or ~ 10^5^ cells) and the low norovirus infectious dose of ~ 100 genome equivalents per subject highlight the risks associated with human exposure to virus-contaminated water and shellfish weeks after the contaminating event (Ettayebi et al., 2016; Teunis et al., 2020).

The design of the experiment sought to improve the robustness of the methodology applied in previous studies. Firstly, three biological replicates were used for each time point and temperature, for which ten technical replicates were used for the infection of the HIE cell culture. Through this extensive use of replicates, we addressed the known biological variability and limited sensitivity previously reported on the norovirus HIE cell culture system. Secondly, given the uncertain relationship between the norovirus titre of the inoculum and subsequent level of replication within the HIE, we classified a quantitative increase of concentration (i.e. GC/mL) to discrete categories of infectious and non-infectious to improve the fidelity of the results. Thirdly, the experiment was designed so that all timepoints were simultaneously tested to avoid freeze–thaw cycles of samples. Combined, this approach represents a very robust methodology to test human norovirus persistence in a water environment.

The infectivity decay rate constants of 0.07 day^−1^ for 4 °C and 0.17 day^−1^ for 16 °C from our study are similar to decay rates from previous norovirus studies using HIE culture. Kennedy et al. (2022) determined that decay rate constants of infectious norovirus varied from no significant decay to 2.2 day^−1^ in filter-sterilised creek water at 15 °C (noting that there was no significant difference identified at 20 °C). Similarly, k values of 0.08 to 0.11 day^–1^ with persistence up to 28 days at room temperature were determined for lake water, drinking water and ultrapure water (Shaffer et al., 2022) and persistence up to 35 days in seawater (Desdouits et al., 2022). Combined, the inferred decay rates from studies using HIE culture support earlier work on volunteers exposed to norovirus where it was showed that infectious norovirus persisted for at least 61 days in groundwater and was able to cause norovirus symptoms (Seitz et al., 2011).

In contrast to the infectivity results, norovirus concentrations determined by direct RT-ddPCR remained constant throughout the experiment. These results again demonstrate the discrepancy between signal reduction quantified by direct molecular methods and viral infectivity, which emphasises the challenges when evaluating the risk of norovirus contamination (Desdouits et al., 2022; Kennedy et al., 2023; Shaffer et al., 2022).

To evaluate the effect of temperature on the persistence of infectious human norovirus, 16 °C was selected to represent the average daily seawater temperature during Autumn (Fall) to Spring in a temperate region (i.e. the upper North Island region of New Zealand; source: https://www.seatemperature.org/) and would represent estuarine water conditions in shellfish growing areas. A temperature of 4 °C was used as a minimal physical deterioration control. Our results did identify a significantly lower decline of infectivity at 4 °C. This is consistent with findings of other studies on enteric viruses such as rotavirus or enterovirus, where an increase of temperature was generally associated with an increase of decay (Boehm et al., 2019). A Japanese study by Takahashi et al. (2016) however found no significant difference between winter and summer in the persistence of the surrogate murine norovirus in estuarine waters.

While this study demonstrates how HIE culture can be utilised to deliver quantitative data, future experiments may be improved by adding further tested time points. Despite the use of artificial water to minimise complexity of the factors potentially effecting infectivity, the variability of the HIE remained considerable, which, along with the low sensitivity, remains a major limitation of the HIE method to detect and quantify infectious norovirus.

Future studies would include investigating further parameters that influence norovirus infectivity in the environment. For our study, sterile artificial estuarine water was chosen to provide a reproducible matrix, particularly for brackish waters that have variable properties compared to seawater. Indeed, other studies have shown that decay rates are highly affected by the matrix (Desdouits et al., 2022; Kennedy et al., 2023; Takahashi et al., 2016). Parameters to consider would include effects of biological (e.g., indigenous microbes), chemical (e.g. phosphates, ammonium), and physical (e.g. temperature, light) factors expected to influence viral decay rates, but are often not controlled for, in virus persistence studies (Pinon & Vialette, 2018). Also, differences in persistence between the various norovirus genotypes are yet largely unknown.

In summary, our study provides relevant data for future persistence and transportation studies, as well as contributing to the improvement of quantitative risk models for environmental transmission from sewage effluents or overflows, for example, into shellfish and their growing waters.
